# A corpus-assisted discourse study of Chinese university students' perceptions of sustainability

**DOI:** 10.3389/fpsyg.2023.1124909

**Published:** 2023-02-16

**Authors:** Ruihong Huang

**Affiliations:** School of Foreign Studies, Shanghai University of Finance and Economics, Shanghai, China

**Keywords:** sustainability, perceptions, Chinese university students, ecolinguistics, corpus, discourse

## Abstract

Education for sustainable development (ESD) in the higher education context plays a critical role in advancing the cause of sustainable development. However, previous research on university students' perceptions of sustainable development is limited. This study used a corpus-assisted eco-linguistic approach to investigate students' perceptions of sustainability issues and responsible actors to address these issues. This quantitative and qualitative study is based on a corpus of 501 collaborative essays on sustainability written by ~2,000 Chinese university students collected with their permission. The results show that the students had a comprehensive perception of the three dimensions of sustainable development. Environmental issues have received the most attention from students, followed by economic and social issues. With regard to perceived actors, students were inclined to view themselves as active participants in the cause of sustainable development, rather than as observers. They called for coordinated action of all relevant parties, such as the government, business sectors, institutions, and individuals. On the other hand, the author also noticed a tendency toward superficial green talk and anthropocentrism in students' discourse. This study aims to contribute to sustainability education by integrating findings into English as a foreign language (EFL) classes. Implications for sustainability education in the context of higher education are also discussed.

## 1. Introduction

Sustainable development concerns the relationship between human beings and nature with different priorities at different stages. The 17 UN Sustainable Development Goals and 169 targets announced in 2015 set a global agenda for the following 15 years. However, as pointed out by Sterling (2016, p. 210), this is an agenda of what we should do in the biophysical world. The recommended actions could only alleviate the current problems of this unsustainable world. If we really want to eliminate those problems, we need to find out the causality, which is believed to be our inner world (i.e., our beliefs, values, and lifestyles). This is where education should play an important role, especially in bringing about lasting changes, or a transformation, because “it is owned and affected by participating stakeholders and learners” (ibid, p. 211). Education does not only help raise the awareness of the youth regarding sustainability issues but also teach them the skills to implement sustainable development. The value of education for sustainable development is emphasized in The Decade of Education for Sustainable Development 2005–2014 (United Nations Educational, Scientific, and Cultural Organization (UNESCO), 2020), an initiative by the United Nations to introduce education for sustainable development (ESD).

Universities play an important role in educating “societies' future decision-makers” about sustainable development (Barth and Timm, 2011, p. 13). M'Gonigle and Starke (2006) argued that universities could promote sustainability “in ways no other modern institution could match” (p. 170). Stakeholders in institutions of higher education involve staff, faculty, students, funding bodies, employers, and the community (Yuan and Zuo, 2013). The success of sustainable development education depends on the participation of all those involved. While many previous studies have examined the beliefs, attitudes, and understandings of lecturers (e.g., Reid and Petocz, 2006; Cotton et al., 2007; Sammalisto et al., 2015), university presidents (e.g., Wright, 2010) and facilities management directors (e.g., Wright and Wilton, 2012), students' beliefs about sustainability have not received enough attention and focus (Aleixo et al., 2021). Students are not passive receivers of instruction or mere outcomes of education. With their unique knowledge and perspectives, they are important participants in the process and producers of the outcomes as well (Levin, 2000). Their engagement is essential to the success of sustainability education. Therefore, it is important to undertake research that focuses on students' perspectives on sustainability issues.

Several previous studies have surveyed students' perceptions of, or attitudes toward, sustainability. Emanuel and Adams (2011) compared students from two American states with regard to their perceptions of campus sustainability. Barth and Timm's (2011) survey of German university students revealed that students were mostly concerned with environmental or ecological issues, followed by social and generational justice. In terms of program intervention, students who studied sustainability in their major tended to focus on technology and economic efficiency and emphasized the role of the state and industry, while those who minored in sustainability tended to focus on consumption behaviors and stressed the role of individuals in sustainability. Yuan and Zuo (2013) surveyed Chinese students' awareness of sustainability issues and their perceptions of key factors for sustainable development within the context of higher education. Eagle et al. (2015) investigated an Australian university's incoming business students' knowledge of and attitudes toward sustainability issues. Aleixo et al.'s (2021) survey of 1,257 students in Portuguese higher education institutions showed that most students realize the importance of sustainable development and advocate reusing and recycling practices to combat climate change. In these previous studies, questionnaires and interviews were commonly used to investigate the subjects' perceptions of sustainability. However, since the participants of the studies were voluntary students, their self-selection might have affected the results of the survey. In addition, the use of categorized questions rather than open questions in the research might neglect the real attitudes or concerns of students, as pointed out by 2011 (2011, p. 21). A notable exception is a survey by Zeegers and Clark (2014) about students' perceptions of ESD, where their survey data were complemented with students' reflective journals; however, only 34 students were involved.

In contrast to the approach of eliciting data from subjects through questionnaires or interviews, one of the strengths of a corpus linguistics approach is its use of large amounts of authentic data that are produced by subjects in what can be considered natural communicative contexts. Corpus-based discourse analysis has been widely used to address social problems such as gender inequality, racism, nationalism, and, recently, environmental issues by revealing the ideology or cognition manifested in the language of the discourse of relevant parties.

In the present case of this study, 501 collaborative textual discourses on sustainability written by ~2,000 students at the Shanghai University of Finance and Economics (SUFE), as part of their coursework in a compulsory College EFL class, were collected and analyzed with the aim to address the following research questions:

1. What are the perceptions of Chinese university students vis-à-vis the environmental, economic, and social dimensions of sustainability?
2. What are the sustainability issues that students consider important in each dimension?
3. According to students, who should take responsibility for sustainable development, and what actions need to be taken?

## 2. Literature review

In this section, I review the terminology and relevant literature useful for understanding the concept of sustainability in the analysis and then present an overview of the integration of corpus linguistics, critical discourse studies, and ecolinguistic approach.

### 2.1. Dimensions of sustainability

Despite widespread use, sustainability is a contested concept with different interpretations over history. The most significant definition of sustainable development is provided in the Brundtland report *Our Common Future*, which defines it as a development “that meets the needs of the present without compromising the ability of future generations to meet their own needs” (World Commission on Environment Development, 1987). The publication of this report in 1987 helped popularize the concept of sustainable development. Since then, this concept has evolved significantly (Bina, 2013; Spindler, 2013). This three-pillar model is a commonly accepted sustainability model, where the roof of sustainability is supported by three pillars: environment, economy, and social equality (Spindler, 2013). These three pillars are not independent of each other. Instead, they tend to be intertwined. For the convenience of discussion, this study describes them separately.

Environmental sustainability has usually been prioritized by scientists. Some early definitions of sustainability mainly focus on environmental sustainability. For example, sustainability was defined, by some, as the “maintenance of natural capital” (Goodland, 1995, p. 10), or more specifically, the improvement of human wellbeing “by protecting the sources of raw materials used for human needs and ensuring that the sinks for human wastes are not exceeded, in order to prevent harm to humans” (ibid, p. 10). In the OECD Environmental Strategy for the First Decade of the 21st Century (OECD, 2001), four criteria are proposed for environmental sustainability: regeneration, substitutability, assimilation, and avoiding irreversibility. Similarly, to help environmental professionals to operationalize the concept of sustainable development, Morelli (2011) has summarized five categories of guiding principles for societal needs, namely preservation of biodiversity, regenerative capacity, reuse and recycling, constraints on non-renewable resources, and waste generation.

The definition of economic sustainability varies according to the approach and perspectives of sustainability. When the issue is approached from the inside, i.e., the organization in business contexts, the concept involves the efficiency of production and economic growth. From the outside perspective of the stakeholders in non-business contexts, it concerns how the activities of the organization influence society over time (Jeronen, 2020). As a pillar of sustainability, economic sustainability generally takes the outside approach, which requires that the production system not only provides people with what they need in an efficient way but also considers the needs of future generations regarding natural resources. It has three criteria: equity of allocation of resources, the efficiency of the use of resources, and the scale of the human economic subsystem (Goodland, 1995, p. 3).

Similarly, although the social dimension of sustainability is well-known, its exact meaning is elusive. There is no uniform definition of this concept (Dempsey et al., 2011). For some, it “requires that the cohesion of society and its ability to work toward common goals be maintained. Individual needs, such as those of health and wellbeing, nutrition, shelter, education, and cultural expression should be met” (Gilbert et al., 1996, p. 12). For others, the goal of a sustainable society is to achieve “fairness in distribution and opportunity, adequate provision of social services,” “gender equity, and political accountability and participation” (Harris and Goodwin, 2001, p. xxix). These different aspects of social sustainability are reflected in four principles of social sustainability: “human wellbeing, equality, democratic government, and a democratic society” (Caradonna, 2014, p. 13).

Different dimensions, or different aspects of sustainability, may highlight different priorities in various contexts. For example, Dempsey et al. (2011) identified social equity and the sustainability of the community as the core of social sustainability within the context of urban social sustainability. It is therefore important for us to know, among the three pillars of sustainability, which dimension is prioritized by students and what major issues in each dimension are matters of concern for them. Thus, universities and teachers could address the issues relevant to students and bridge the gap in sustainability literacy, as well.

### 2.2. Corpus-assisted discourse studies and ecolinguistics

Discourse is a complex concept that involves an interaction between language and society. In linguistic terms, discourse is the language above a sentence level or language in use. In social science, it relates to “the social process of communication” to build relationships and improve understanding (Lemke, 1995, p. 6). Thus, discourse analysis encompasses a collection of theories and methods to investigate communicative activities, including the use of language in contexts, from various disciplines, such as linguistics, communication, and anthropology, with an aim to address communication and social problems (Gordon, 2015). Because of the integration of methods from many fields, Van Dijk (2015, p. 466) suggests using “discourse studies” instead of “discourse analysis.” Through discourse analysis, language is studied as a means of communication. Meanwhile, since language can reflect and shape our perception and mind, it can also serve as “a method of inquiry” (Tracy, 2005, p. 726).

Corpus linguistics is a methodology of linguistic study that relies on large amounts of authentic data to unveil the regularity of language use, or patterns, with the aid of software. Both corpus linguistics and discourse analysis require authentic data. The term Corpus-Assisted Discourse Studies or CADS was first coined by Partington (2004). It is defined as “that set of studies into the form and/or function of language as communicative discourse, which incorporates the use of computerized corpora in their analyses” (Partington et al., 2013, p. 10). Major techniques and procedures in corpus linguistics, such as *keywords, associate sets, word lists*, and *concordance*, are used to retrieve quantitative information about lexical items and reveal meaningful patterns of language use in the discourse under study (Scott and Tribble, 2006; Scott, 2016). Using corpus in discourse analysis enables researchers to reduce their cognitive bias and shed light on how speakers or writers draw on language resources to construct discourse and present different views of the world (Baker, 2006). Thus, in contrast to conventional corpus linguistics, CADS aims to reveal some truth of the world as manifested in language rather than regularities of the language pattern in themselves. Topics such as immigration and gender inequality have been on the agenda of researchers' focus on discourse studies for a long time. Climate change and environmental or ecological issues have received a lot of attention in the past 20 years, which spurred the emergence of ecolinguistics.

Ecolinguistics treats language as part of the ecological system and highlights its influence on the whole ecological system beyond human beings, which includes plants, animals, and the environment we live in, and also takes into account future generations (Stibbe, 2021). As a form of discourse analysis, in ecolinguistics, texts are studied to uncover “the hidden stories that exist between the lines” (ibid, p. 2). According to Stibbe (2004), these stories indicate how our thoughts, beliefs, perceptions, and ideologies are manifested in our discourse, and they could shape the mindset of young people. Once stories are discovered, we could critique or promote them to build a more ecological world. In the Chinese context, Huang and Zhao (2021) proposed harmonious discourse analysis as an approach to diversify ecolinguistics with inspiration from traditional Chinese philosophy, seeking to provide a more holistic view of the harmonious relationship between human beings and the world.

Since the advent of ecolinguistics in the 1990s, scholars have explored different levels of language representation, such as vocabulary, syntax, semantics, metaphor, and discourse. This study follows the analytic framework proposed by Stibbe (2014, p. 118) to investigate “how clusters of linguistic features come together to form particular worldviews.” Stibbe (2021, p. 6) proposed eight types of stories or “cognitive structures” that are shared by people in a community or culture, which also shape our behaviors. They include ideologies, framings, metaphors, evaluations, identities, convictions, erasure, and salience. In this study, the author mainly focuses on ideology and salience. The exploration of how the world is, and ought to be, in the minds of Chinese college students and what aspects of daily life are regarded as important or worthy of attention is done by analyzing the linguistic features in their discourse using a corpus-assisted approach.

## 3. Materials and methods

### 3.1. Corpus

The corpus consists of 501 essays with a total of 1,012,409 tokens on the theme of sustainability and innovation: human, environment, economy, and development of technology. These essays were written by first-year undergraduates at SUFE as part of coursework in the Academic English Course from 2018 to 2019. Each essay was produced in the form of collaborative writing by four to five students. This writing assignment was also part of a broader academic project participated in by numerous Chinese universities. From 2015 to 2020, the China English for Academic Purposes Association (CEAPA) held the International Collegiate Conference, where annually, college students presented works on a given theme in English. This project aimed to improve university students' academic English skills while raising their awareness of social responsibility through problem-solving projects. Many universities joined this event by integrating the writing project into their EFL classes, selecting candidates through campus-level competitions, and recommending candidates to attend the International Collegiate Conference. The theme of the conference for the years 2018 and 2019 was sustainability and innovation, with a focus on the 17 UN Sustainable Development Goals.

### 3.2. Procedures for corpus analysis

Corpus techniques that are used in this analysis include keywords, collocate, and concordance. In corpus linguistics, keywords refer to the words in a text or a corpus that are unusually more frequent than in the reference corpus (usually a corpus of general English). The British National Corpus was used as the reference corpus in the study as it has a large size (~100 million words), and the well-balanced design makes it a widely accepted corpus representing general English. Keywords are especially useful in revealing the aboutness or style of the text (Scott and Tribble, 2006). Wordsmith (Version 7) was used in the present study to extract keywords and key keywords (i.e., keywords that have a wide dispersion) (Scott, 2016). After the keywords were extracted, the researcher also explored the semantic network of these keywords through their Associate Set and ran concordance searches for the keywords to examine the co-text of these keywords. Meanwhile, the study also used #LancsBox 6.0 (Brezina et al., 2021) to present the network of some keywords. The collocate function of Wordsmith was used to identify the subject of the obligatory modal verb *should*, which may indicate who should take responsibility for sustainability development. Studying the salience patterns realized in language, which, in this case, included keywords and typical collocations, gave a glimpse into the stories in students' minds, i.e., what sustainability issues are regarded as significant and noteworthy.

## 4. Results

### 4.1. Major sustainability issues for students

The default setting of the Wordsmith was used to extract the keywords: The minimum frequency was set at 3, the minimum percentage of text was set at 5%, and the minimal log ratio was set at 1.5. In our corpus of ~1 million words, 1,171 keyword entries were found. Appendix A provides the top 40 keywords ranked in order of keyness of log-likelihood. Log-likelihood is a measure of the keyness in terms of statistical significance; the log ratio deals with the effect size of keyness.

For the convenience of discussion, the study uses the commonly accepted three-pillar model of sustainability as the framework of analysis. As can be observed from Table 1, the nominal and adjective forms of the terms of the three dimensions of sustainability are all keywords. The keyness of *environmental* and *environment* is much higher than that of *economy, economic, social*, and *society*, which suggests that environmental issues are a major concern for students, followed by economic issues and social ones.

**Table 1:** Statistics of keywords related to the three sustainability dimensions.

**Keyword**	**Freq**	**%**	**Texts**	**RC. Freq**.	**RC. %**	**Log_L**	**Log_R**	***P***
Environmental	1,194	0.12	202	8,411		3,937.76	3.80	< 0.001
Environment	1,283	0.13	314	12,935	0.01	3,441.10	3.28	< 0.001
Economy	803	0.08	204	10,365	0.01	1,819.30	2.93	< 0.001
Social	1,232	0.12	328	41,744	0.04	993.69	1.54	< 0.001
Economic	781	0.08	254	23,376	0.02	758.17	1.71	< 0.001
Society	697	0.07	273	22,457	0.02	607.83	1.61	< 0.001

RC, reference corpus; Log_L, log-likelihood; Log_R, log ratio. The default p-value is 0.000001.

Table 1 summarizes the statistical data about the comparison between the total frequencies of each of these keywords in the students' discourse corpus with that of the reference corpus. However, it is not clear to what extent these keywords spread across the corpus. To solve this problem, the study employs the key keyword function of Wordsmith. A “key keyword” is a word that is key in more than one of a quantity of related texts (Scott, 2016). A total of 666 key keywords were obtained from the database by setting a “key keyword” to occur in minimum 5 texts with at least 10 keywords per text. That is to say, 666 keywords occur in more than five texts. As shown in Table 2, of the three dimensions of the concept of sustainability, *environment(al)* is the most salient in the student discourse corpus. Altogether *environmental* occurs as a keyword in 79 texts and *environment* in 71 texts.

**Table 2:** Associate sets of sustainability keywords.

**Environmental (79)**	**Environment (71)**	**Economy (32)**	**Economic (22)**	**Social (25)**	**Society (14)**
Environment	Environmental	Development	Economy	Inequality	Men
Protection	Pollution	Sustainable	Development	Women	Women
Pollution	Protection	Economic	Sustainable	Men	Inequality
Questionnaire	Questionnaire	Resources	China's	Gender	Gender
Recycling	Development	More	China	Society	Social
Packaging	Plastic	China	Industry	Equal	Discrimination
Materials	Sustainable	Environment	Growth	Equality	Equality
Waste	Our	Sharing	Resources	Rights	Males
Development	We	Industry	United	Students	Equal
Garbage	Ecological	Shared	Investment	Groups	Status
Friendly	Carbon	Environmental	Transportation	Family	Women's
Energy	Waste	Innovation	Trend	Level	Male
Sustainable	Garbage	China's	Living	Discrimination	Employment
Consumption	Respondents	City	Industries	Self	People's
Recycle	Questionnaires	Government	City	Development	Phenomenon
People's	Recycling	Energy	Products	Income	Survey
Industry	Shanghai	Questionnaire	Innovation	Feminist	Problems
Our	Students	Behavior	Consumption	Wechat	Students
Awareness	China	Living	Labor	College	Development
Plastic	Energy	Industrial	More	Promote	Questionnaire

The number in the brackets indicates the number of texts in which the word is a keyword.

Table 2 presents the associates of each of the six keywords related to the three major dimensions of sustainability in order of the association of strength. Associates are the keywords closely connected with a key keyword in the same text (Scott, 2016). The strength of association was measured statistically using the MI3 score in our study. The minimum associated texts in which a keyword co-occurs with the key keyword under study were set to 3.

#### 4.1.1. Environmental dimension

As indicated by the associates of *environment* and *environmental* (see Table 2), the environmental issues that received the most attention include pollution, protection, recycling, packaging, carbon, waste/garbage, and energy. In their writing, students aimed to raise people's awareness about these environmental issues, call for appropriate governmental policies, and press for the adoption of a lifestyle that would favor the environment.

Among the associates of *environment*(*al*), *garbage* is worth special attention. As can be observed in Appendix A, the term's keyness is particularly high, ranking second of all the keywords. There is a great overlapping of associates between *environment, environmental*, and *garbage* (see Tables 2, 3). The associate set of *garbage* highlights the recycling of garbage (waste or rubbish) on campus in Shanghai, or in China, particularly the large quantities of plastic garbage or packages caused by the fast-growing express delivery service. Students were concerned about the current environmental situation and the damage caused by garbage. Sorting or classification of garbage for recycling was proposed as a promising solution to the problem. Both government and individuals, especially students, were perceived to have a role to play in tackling the garbage issue.

**Table 3:** Top 20 associates of *garbage*.

***N***	**Associate**	**Strength**	**Texts**	**%**
1	Classification	13.17	33	58.00
2	Waste	12.66	35	62.00
3	Recycling	12.27	33	58.00
4	Questionnaire	10.96	38	67.00
5	Classify	10.92	15	26.00
6	Packaging	10.39	16	28.00
7	Students	10.12	30	53.00
8	Disposal	10.12	13	23.00
9	Pollution	10.12	19	33.00
10	Environmental	10.09	21	37.00
11	Shanghai	9.94	23	41.00
12	Trash	9.86	11	19.00
13	Campus	9.76	19	33.00
14	Delivery	9.76	14	25.00
15	Sorting	9.56	10	17.00
16	Plastic	9.42	12	21.00
17	Classified	9.37	9	16.00
18	Express	9.36	12	21.00
19	Questionnaires	9.26	19	33.00
20	Recyclable	9.23	9	16.00

Another interesting associate of *environment/environmental* is *Ant Forest*. A total of 305 occurrences of the item *Ant Forest* were found in the corpus. The Ant Forest Initiative is an environmental initiative launched by the Ant Financial Service Group, an online payment company affiliated with Alibaba. This program started in 2015 with an aim to mobilize people, especially its app users, to monitor their carbon footprints and live a more environmentally friendly lifestyle. Through an Ant account, users earned virtual-energy points for their behaviors to reduce carbon emissions, such as using an online payment service, walking instead of driving, and so on. When one earns enough points, the company and its partners will grow a real tree in one of the deforested areas in China. This initiative was awarded “Champions of the Earth” in 2019, a United Nations' environmental honor to recognize outstanding initiatives to protect the environment. From the students' writing, we can see that this initiative was well received among the youth in China.

#### 4.1.2. Economic dimension

**Figure 1:**
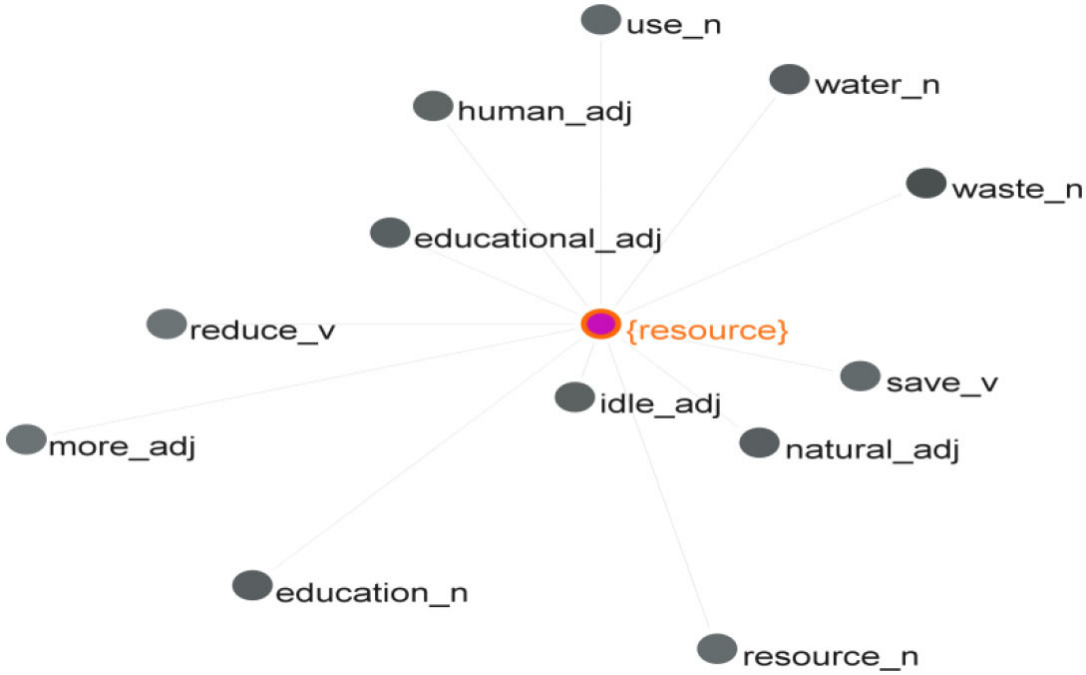
Significant collocates of *resource*.

*Economy* or *economic* is closely associated with *development, sustainability, resources, China, sharing, industry, innovation*, and *energy*, among others (Table 2). The word *resources* have a strong association with *economy*/*economic*. Using GraphColl of #LancsBox 6.0, the study obtained the graph of the significant collocates of *resource*, as shown in Figure 1, by setting its span to five words to its left, the collocation statistic value threshold of MI at 3.0 and minimum frequency of its collocates at 25. This graph presents the network of the adjective, noun, and verb collocates of the word *resource*. The closer a collocate is to the search word *resource* in the center, the stronger its collocation strength is. For example, the word *idle* is a stronger collocate of *resource* than *waste*.

According to the Oxford Dictionary, a resource is “a supply of something that a country, an organization, or a person has and can use, especially to increase their wealth.” From the lexical network of the word *resource*, we can see that students are especially concerned about the proper use of idle resources, natural resources, and water resources. Based on the concordance lines where *resources* co-occur with *economy/economi*c, it was observed that students suggest a positive relationship between the proper use of resources and sustainable economic development. They embrace a green economy through the sustainable use of natural resources, advocate the development of sharing economy through exploiting idle resources to improve efficiency, and seek to improve economic gains from reduced consumption of resources (such as land and materials) and recycling. For example, students wrote:

1. There is a great number of idle resources in various fields. If the sharing economy can use these resources efficiently, its depth and breadth will be expanded.
2. Through the sustainable utilization of natural resources, green economic development can improve the utilization and regenerative ability of the natural environment to the greatest extent.
3. Garbage classification collection can reduce the amount of garbage treatment and treatment equipment, reduce the cost of treatment, and reduce the consumption of land resources, with social, economic, and ecological benefits.

At the same time, from the student discourse, we can see that while the natural world, including human beings, is framed as a kind of resource, its inherent value is diminished. Being idle is considered not optimum. We could also ascertain anthropocentrism common in many students' discourses and get a sense of overconfidence in the human ability to harness nature, as shown in example (2).

In addition to promoting the practice of saving and making full use of natural resources to optimize human interest, the students also press for an equitable allocation of resources for personal growth and wellbeing. They were aware of the social problems caused by the unequal distribution of resources, as shown in (4) and (5). They argued that equal access to economic and educational resources is essential to eradicating gender discrimination and other social inequalities. Providing opportunities to the poor and good education can help close the gap between the poor and the rich. They suggested that people's right to equal resources should be protected by law [see (6)].

(4) It is a common phenomenon that people prefer bigger cities where there are more resources like job opportunities or education, and as a result of this, bigger cities are usually much more crowded than smaller ones, and it also means higher housing prices. This is what we call uneven distribution of resources.
(5) On the contrary, the students with low social and economic status have no more resources to make up for their previous academic failures, and the risk avoidance motivation of the decline in social status is relatively low, which leads them to be in a very big disadvantage in the access to higher education.
(6) We should ensure that women enjoy equal opportunities and resources for economic development in accordance with the law.

#### 4.1.3. Social dimension

With regard to the associate set of *social* and *society*, the (in)equality issue is highlighted in students' discourse, especially gender inequality, as illustrated in Table 2. The associate set of *gender* was further explored in the study. Apart from commonly connected words such as *women, men, female*, and *male*, words that point to the concerns of students over gender inequality issues include (*in)equality, discrimination, work(place), job*, and *employment*, among others. It can be observed that students were especially concerned about equal opportunities for men and women in the job market and discrimination against women in the workplace. This may be because the proportion of female students at the author's university is very high (the ratio between male and female graduate students in 2018 is ~1:1.7), and the job market in China is very competitive.

In addition to gender (in)equality, health or the mental wellbeing of university students and access to quality education were also emphasized. Human wellbeing, which is related to self-identity, self-esteem, self-actualization, self-efficacy, and self-learning, has received much attention from students. A strong association of *social* that is worth mentioning is *self* (Table 2). For example, students commented that:

(7) The popular trend of the “Kua Kua group” provided a platform for university students to express their feelings as well as build up their own self-esteem and social circles through communication with others.
(8) Ant forests have met the needs of users for entertainment, self-identity, and social interaction to many degrees.

Another important associate of *social/society* is *education*. In the associate set of *education*, some words are naturally connected with education, such as *student, school(s), teaching, learning, teachers*, and *class*. The list highlights *quality, high, university/universities*, and *college*. This indicates that students are most concerned about higher education and high-quality education.

### 4.2. Perceived actors and their responsibilities

What are the views of students with regard to who is to assume the responsibility for sustainable development and what actions need to be taken? One clue of language lies in the collocates of *should*, one of the most frequent words in the corpus. The modal auxiliary s*hould* have 2,230 tokens in the corpus and widely spreads out in the whole set of texts with a dispersion of 0.95. It ranks 46th in terms of frequency among 21,325 different words. Semantically and pragmatically, *should* is generally used to express obligation or necessity. Its left collocates may imply the actors, while the right collocates may indicate actions to be taken. The collocates of *should* were searched for within the span of one word to its left and one word to its right. As presented in Table 4, governments (including countries and departments), business sectors (such as companies and enterprises), institutions (such as schools and universities), individuals (such as everyone and people), or groups (such as women and students) were perceived as important actors.

**Table 4:** Top 15 collocates of *should* within a span of one word to its left and one word to its right.

**L1 collocate**	**Freq**.	**R1 collocate**	**Freq**.
We	404	Be	714
Government	173	Take	82
They	116	Pay	70
It	62	Have	67
Students	60	Make	60
Women	35	Do	48
Companies	29	Strengthen	43
Enterprises	28	Improve	32
School	25	Focus	28
Schools	25	Provide	26
People	23	Set	28
Universities	20	Give	26
Everyone	15	Increase	21
Countries	14	Try	19
Departments	14	Put	19

**Figure 2:**
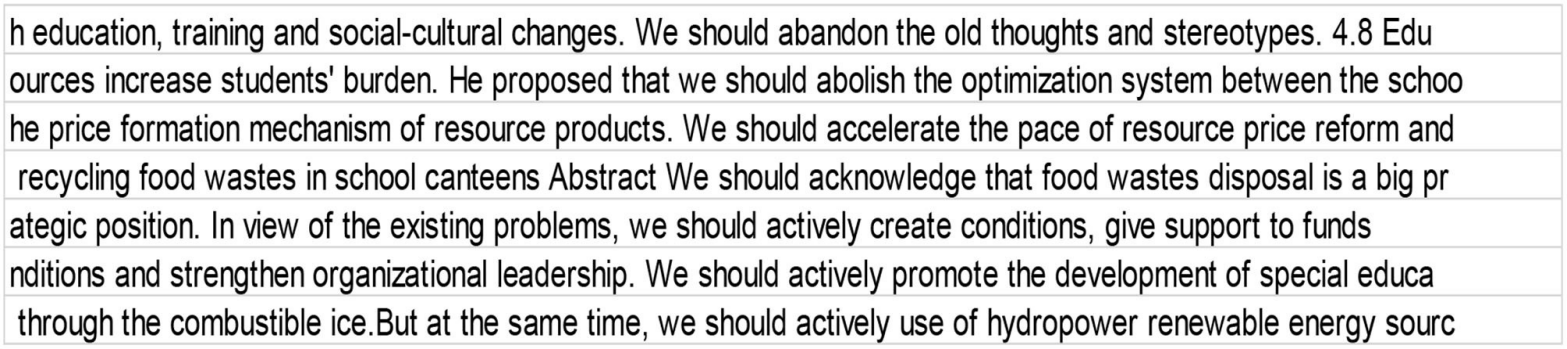
Extracts from the concordances of *we should*.

In addition, pronouns, such as *we, they*, and *it*, are also at the top of the left collocate list of *should*. A close examination of these words in context revealed that the pronoun *they* mainly refer to the relevant actors or stakeholders, as mentioned earlier, while *it* is used to refer to some phenomenon or behavior, or stakeholders such as the government or a specific company. What is noteworthy is the use of *we*. The meaning of the collective pronoun *we* is complex. It may include the reader/listener or exclude them (Mulderrig, 2011). In other words, it may refer to the student writers only (i.e., exclusive *we*) or include both the writer and the reader (i.e., inclusive *we*), or even the other relevant stakeholders. Of the 404 occurrences of *we should*, only five indicate exclusive usage, three are ambivalent, and the rest are inclusive. The inclusive *we* assume a consensus with the readers and other stakeholders. It has a sense of solidarity. Instead of directly requesting others to do something, the student writers were inclined to call for coordinated action, which is hard for all relevant actors to refuse. Figure 2 is an extract from the corpus concordances of *we should*.

As presented in Table 4, the verb collocates of *should* with a span of one word to its right comprises primary verbs such as *have, be*, and *do* that can be used as lexical verbs or auxiliary verbs and lexical verbs such as *take, pay, make*, and many others. As the meaning of *be* is not clear without context, the researcher further examined the three-word clusters of *should be* +verbs. The most frequent clusters include *should be taken, should be paid*, and *should be made*. For example, *measures should be taken, (something) should be taken into account, (something) should be paid attention to*, and *efforts should be made*. Comparing these clusters with the top verbs that accompany *should*, there are great overlaps of the main verbs such as *take, pay*, and *make*. For example, students suggested that:

(9) Perhaps, the government *should take* the responsibility to make new policies that promote the upgrading of the domestic building material market.
(10) From this, we come to the conclusion that enterprises *should pay* attention to product quality and technological innovation.

The common collocates of *should make* include *efforts, use of policy*, and *law*, indicating the necessity of efforts and regulations. *Should have* is often followed by nouns such as *right, opportunities, choice*, and *awareness*. For example:

(11) Women should have opportunities to go to work, to explore their potential, to cultivate their core competitiveness, and to develop their leadership of decision-making in all respects of the public and private life.

These concordance lines suggest that students believe that the commitments of different parties and the consensus of the whole society are required if we want to make a difference in this unsustainable world.

## 5. Discussion and conclusion

These results indicate that the students at SUFE have a comprehensive and sophisticated understanding of the concept of sustainability. The themes of their writing covered various aspects of sustainable development, including the environment, society, economy, culture, interaction, technology, innovation, and so on. The three major sustainability dimensions, i.e., environment, society, and economy, have received great attention. Among them, the environmental or ecological dimension of sustainability was prioritized by the students. This tendency is much in line with the findings of some other scholars, such as those of Barth and Timm's (2011) investigation of a German university, Yuan and Zuo's (2013) survey of a Chinese university, and Fisher and McAdams' (2015) survey of an American university. It is interesting to note that the students under study were especially concerned about the garbage disposal, recycling, and reducing waste. These issues were also regarded as important factors in environmental sustainability by the student participants in Yuan and Zuo's (2013) survey. However, we noticed that despite the criticism of shallow environmentalism by Stibbe (2004) about two decades ago, the students' textual discourse still shows a feature of shallow environmentalism characterized by focusing on immediate ecological problems and solutions while overlooking the deep “psychological causes” (p. 243).

The second most important dimension that has drawn great attention from the students is economic sustainability, which is different from Barth and Timm's (2011) results that social and generational justice followed environmental aspects as the second important dimension for the students in a German university. This difference may be due to the influence of China's fast economic development and the great importance that the whole society attaches to economic prosperity. Another possible reason could be that most students at SUFE have economics or business-related majors. As the economic dimension is closely related to the environmental aspect (Goodland, 1995), students were concerned about the efficiency of their use. They were attracted by new models such as the green economy and sharing economy.

As for the social dimension, gender inequality at the workplace and in job markets was made salient in the students' discourse. Previous studies suggested that gender might affect how students perceive sustainability (Fisher and McAdams, 2015). Future research that takes into account the gender factors of the students under study would help to shed important light on the issue. Keywords analysis helps us identify the salient concerns of students. Meanwhile, we should also be aware of what is erased, such as the other two principles of social sustainability: democratic government and democratic society.

Concerning the students' perception of responsible actors, the results suggest a strong association with the government (state or country), industry, and individuals, which is in common with the findings of Barth and Timm's (2011) about German students. It is also worth noting that the students were inclined to regard themselves as part of the broader community on the planet and feel a responsibility to participate in sustainable development. Meanwhile, the students' writing indicates that institutions sometimes fail to address the issues that are of significant concern to them, such as the quality of education and the effectiveness of liberal education curricula. It is, therefore, important for universities to develop a shared vision through dialogs with students.

Taken together, in students' writing, we could see their concerns about individual growth opportunities, the relationship between human beings and their environment, and the relationship between human beings. Generally, they have a positive attitude toward sustainable development and are willing to contribute to the cause. Yet, overall the students' discourse in this study is ambivalent discourse (Stibbe, 2021). Behind these words, the researcher identifies some stories of superficial green talk, such as garbage classification and recycling. These practices could help protect our ecosystem. Too much attention on garbage disposal may distract attention from the real cause behind the garbage problem; that is, our understanding and ideology of the relationship between human beings and the world are human-centered. Reading between the lines, we could also see a tendency toward anthropocentrism. According to Huang and Zhao (2021), who based their harmonious discourse analysis on traditional Chinese philosophy, human beings, non-human species, and non-living beings are the basic elements of the ecosystem. Human-orientedness does not mean human benefits should be paramount. All beings need to have a harmonious coexistence. Since human beings depend on natural resources to survive, they have the responsibility to take care of the natural environment.

This corpus-assisted discourse analysis helps to reveal Chinese students' perceptions of sustainable development, as demonstrated in their writings. Yet, the findings of this study need to be interpreted with caution, given the limitations of corpus data. A limitation of the study was the homogeneity of the participants in terms of majors, which may limit the generalizability of these findings to the general university student population in China. Future research should include students in different disciplines from different universities. Another limitation concerns the nature of students' writing. Compulsory collaborative work may create some peer pressure among group members and thus influence their writings, which may not reflect the real opinions of each individual student. Future researchers may want to employ questionnaires or interviews to triangulate these findings.

Despite the limitations, the large authentic data produced by students provide a good starting point for researchers to understand their opinions of sustainability. Moreover, the practice of integrating sustainability education into an EFL course at SUFE demonstrates the possibility of providing sustainability education in seemingly unlikely subject areas. It will be of great benefit for teachers and institutions to learn how this kind of language-oriented learning activity in an EFL class enhances students' awareness and understanding of sustainability issues. As noted earlier, the students are aware of their roles in taking care of the environment. Teachers can use this finding as a point of departure and encourage students to rethink the relationship between humans and nature. Positive discourses from their local culture and traditional Chinese culture that embrace a philosophy of a harmonious relationship between human and non-human species and their environment can be used as examples of alternative paths to improve human wellbeing in addition to technological development. Hopefully, this transformation of mindset or cognition will ultimately lead to changes in behaviors.

## Data availability statement

The original contributions presented in the study are included in the article/supplementary material, further inquiries can be directed to the corresponding author.

## Ethics statement

Ethical review and approval was not required for the study on human participants in accordance with the local legislation and institutional requirements. Written informed consent from the participants' legal guardian/next of kin was not required to participate in this study in accordance with the national legislation and the institutional requirements, however, the data was collected with signed consent from the students.

## Author contributions

The author confirms being the sole contributor of this work and has approved it for publication.

## Appendix A

**Table A1:** Top 40 keywords in the corpus.

***N***	**Keyword**	**Freq**.	**%**	**Texts**	**RC. Freq**.	**RC. %**	**Log_L**	**Log_R**	***P***
1	Students	6,502	0.64	369	14,514	0.01	34,081.39	5.46	< 0.001
2	Garbage	1,790	0.18	95	278		14,832.89	9.31	< 0.001
3	Questionnaire	1,992	0.20	385	1,181		14,152.12	7.37	< 0.001
4	China	1,923	0.19	377	4,912		9,659.31	5.27	< 0.001
5	Online	1,238	0.12	232	597		9,080.89	7.67	< 0.001
6	Shanghai	1,095	0.11	257	226		8,864.60	8.90	< 0.001
7	We	9,257	0.91	499	3,00,833	0.30	7,968.10	1.60	< 0.001
8	Recycling	1,202	0.12	105	1,050		7,962.51	6.81	< 0.001
9	Development	2,869	0.28	429	32,010	0.03	7,201.63	3.14	< 0.001
10	Classification	1,124	0.11	84	1,660		6,613.69	6.06	< 0.001
11	Campus	919	0.09	150	625		6,379.15	7.17	< 0.001
12	Our	4,333	0.43	478	93,455	0.09	6,257.40	2.19	< 0.001
13	College	1,701	0.17	259	9,995	0.01	6,143.02	4.06	< 0.001
14	Research	2,375	0.23	460	26,704	0.03	5,930.61	3.13	< 0.001
15	Respondents	934	0.09	243	1,084		5,824.11	6.40	< 0.001
16	Internet	647	0.06	219	97		5,375.40	9.36	< 0.001
17	Education	2,204	0.22	227	25,880	0.03	5,342.50	3.06	< 0.001
18	SUFE	570	0.06	132	0		5,241.40	138.21	< 0.001
19	Gender	968	0.10	108	1,960		5,224.64	5.60	< 0.001
20	Questionnaires	739	0.07	288	490		5,152.39	7.21	< 0.001
21	Survey	1,351	0.13	357	8,055		4,844.99	4.04	< 0.001
22	Sustainable	714	0.07	183	680		4,647.65	6.69	< 0.001
23	Packaging	742	0.07	47	959		4,512.11	6.25	< 0.001
24	Energy	1,477	0.15	153	12,098	0.01	4,486.92	3.58	< 0.001
25	Waste	1,194	0.12	167	6,657		4,420.33	4.14	< 0.001
26	Data	1,625	0.16	398	18,084	0.02	4,086.04	3.14	< 0.001
27	Wechat	431	0.04	95	0		3,963.23	137.80	< 0.001
28	Environmental	1,194	0.12	202	8,411		3,937.76	3.80	< 0.001
29	Sharing	793	0.08	66	2,414		3,753.40	5.01	< 0.001
30	Consumption	829	0.08	159	3,286		3,554.67	4.63	< 0.001
31	People	3,747	0.37	473	11,6,196	0.12	3,458.11	1.66	< 0.001
32	Consumers	734	0.07	145	2,290		3,444.02	4.98	< 0.001
33	Environment	1,283	0.13	314	12,935	0.01	3,441.10	3.28	< 0.001
34	Improve	964	0.10	371	6,171		3,338.68	3.94	< 0.001
35	Bikes	507	0.05	27	457		3,337.55	6.77	< 0.001
36	People's	911	0.09	337	5,597		3,219.82	4.00	< 0.001
37	Chinese	827	0.08	259	4,153		3,210.81	4.29	< 0.001
38	According	1,316	0.13	443	15,686	0.02	3,156.87	3.04	< 0.001
39	Pollution	798	0.08	136	4,107		3,064.46	4.25	< 0.001
40	China's	489	0.05	175	564		3,053.57	6.41	< 0.001
